# Three-dimensional porous hollow fibre copper electrodes for efficient and high-rate electrochemical carbon dioxide reduction

**DOI:** 10.1038/ncomms10748

**Published:** 2016-02-18

**Authors:** Recep Kas, Khalid Khazzal Hummadi, Ruud Kortlever, Patrick de Wit, Alexander Milbrat, Mieke W. J. Luiten-Olieman, Nieck E. Benes, Marc T. M. Koper, Guido Mul

**Affiliations:** 1 PhotoCatalytic Synthesis Group, Faculty of Science and Technology, MESA^+^ Institute for Nanotechnology, University of Twente, Meander 229, P.O. Box 217, 7500 AE Enschede, The Netherlands; 2 College of Engineering, University of Baghdad, P.O. Box 47024, 10071 Aljadria, Baghdad, Iraq; 3 Leiden Institute of Chemistry, Leiden University, Einsteinweg 55, P.O. Box 9502, 2300 RA Leiden, The Netherlands; 4 Inorganic Membranes Group, Faculty of Science and Technology, MESA^+^ Institute for Nanotechnology, University of Twente, P.O. Box 217, 7500 AE Enschede, The Netherlands; 5 Molecular Nanofabrication Group, Faculty of Science and Technology, MESA^+^ Institute for Nanotechnology, University of Twente, P.O. Box 217, 7500 AE Enschede, The Netherlands

## Abstract

Aqueous-phase electrochemical reduction of carbon dioxide requires an active, earth-abundant electrocatalyst, as well as highly efficient mass transport. Here we report the design of a porous hollow fibre copper electrode with a compact three-dimensional geometry, which provides a large area, three-phase boundary for gas–liquid reactions. The performance of the copper electrode is significantly enhanced; at overpotentials between 200 and 400 mV, faradaic efficiencies for carbon dioxide reduction up to 85% are obtained. Moreover, the carbon monoxide formation rate is at least one order of magnitude larger when compared with state-of-the-art nanocrystalline copper electrodes. Copper hollow fibre electrodes can be prepared via a facile method that is compatible with existing large-scale production processes. The results of this study may inspire the development of new types of microtubular electrodes for electrochemical processes in which at least one gas-phase reactant is involved, such as in fuel cell technology.

## Abstract (web summary)

Aqueous phase electrochemical reduction of CO_2_ requires an active electrocatalyst and efficient mass transport. Here, the authors report a hollow fibre copper electrode displaying compact 3D geometry, with a large area, three phase boundary for gas-liquid reactions, and subsequently enhanced performance.

## Graphical abstract

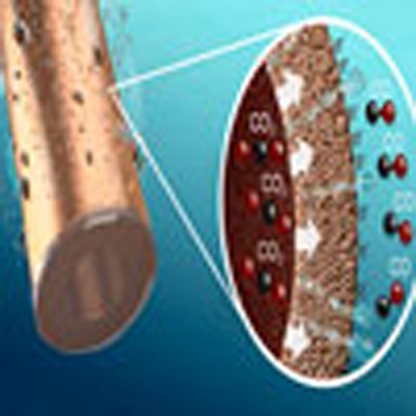

The accumulation of carbon dioxide (CO_2_) in the atmosphere is generally accepted to have a significant impact on (local) climate conditions^1^,^2^,^3^. Immediate measures must be taken to minimize carbon emissions and to mitigate this impact^4^. A promising methodology contributing to reduction of CO_2_ emissions is to electrochemically convert CO_2_ to useful chemicals, while using electricity generated by renewable energy sources^5^,^6^,^7^. Then, an efficient, preferably cheap, and stable electrocatalyst, which can reduce CO_2_ at high current densities, is required^8^. In recent years, significant progress in understanding and enhancing performance of electrodes in electrochemical CO_2_ reduction has been made. For example, formation of carbon monoxide (CO) at low potentials using noble metals in aqueous electrolytes has been reported with high selectivity over hydrogen formation, albeit at low current densities^9^,^10^,^11^. Higher current densities, maintaining CO production selectivity, were obtained by using ionic liquids as electrolyte^12^,^13^. However, practical application of ionic liquids is challenging, as cost is generally high and stability questionable^9^,^14^. An attractive alternative for noble metal electrodes is the use of copper electrodes. Copper electrodes are well known to produce hydrocarbons from CO_2_, with the onset potential (≈0.5 to −0.7 V versus reversible hydrogen electrode (RHE)) depending on the preparation method^15^,^16^,^17^. In general, potentials of −0.8 to −1 V versus RHE are necessary to obtain reasonable faradaic efficiency (FE) for products of CO_2_ reduction on these electrodes. Although less expensive and much more abundant than other CO evolving electrodes, poor activity, selectivity and stability in the formation of CO and formic acid prevent the practical application of polycrystalline copper^18^,^19^. Recently, Li *et al.*^20^ reported production of CO and formic acid with reasonable FE at low overpotentials on copper nanoparticles, when synthesized by electrochemical reduction of copper oxides. At a potential of −0.5 V versus RHE, a partial current density of 2.1 mA cm^−2^ for CO_2_ reduction was obtained, yielding an FE of 35% for CO and 33% for formic acid. Ethylene and methane are other products typically observed in aqueous-phase CO_2_ reduction, which are extensively formed at more negative potentials than −0.5 V versus RHE^21^.

Besides the structural requirements of the copper electrodes, process conditions need to be optimized as well, to allow efficient electrochemical reduction. Inorganic hollow fibres and microtubular electrodes have been applied as component in solid oxide fuel cells. A high power output and low fabrication costs are beneficial properties of such electrode configurations^22^,^23^,^24^. In aqueous electrolytes, hollow fibres composed of nickel and carbon have also been used as cathode for proton or oxygen reduction, respectively^25^,^26^. Finally, microtubular gas diffusion electrodes (GDEs) made of carbon nanotubes have been applied in aqueous-phase electrochemical conversions^27^. Here we demonstrate that Cu hollow fibre electrodes are very efficient in electrocatalytic conversion of CO_2_, owing to their porous structure and excellent mass transport properties. Not only the hydrogen evolution reaction is suppressed on these electrodes to levels not reached previously on copper surfaces to the best of our knowledge, but also the overall current density for CO_2_ reduction is unprecedentedly high at low potentials.

## Results

### Physical characterization of the copper hollow fibres

**Figure 1:**
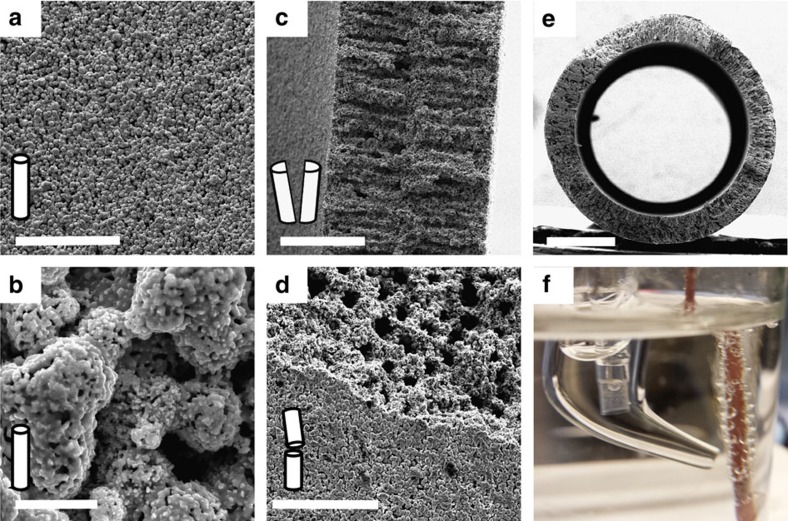
Physical characterization of Cu hollow fibres. (**a**) SEM images of low and (**b**) high magnification of the outer surface of the Cu hollow fibre. Scale bars, 50 and 2 μm, respectively. (**c**) Cross-sectional image of a perpendicularly broken Cu hollow fibre. Scale bar, 100 μm. (**d**) Outer surface and cross-section of a Cu hollow fibre in the parallel direction to the length of the hollow fibre. Scale bar, 50 μm. (**e**) Cross-sectional image of the Cu hollow fibre taken at low magnification. Scale bar, 500 μm. (**f**) Cu hollow fibre employed as an electrode at 20 ml min^−1^ gas flow.

The preparation of metal hollow fibres from nickel and stainless steel has been described in the literature previously^28^,^29^. We adapted this method and prepared Cu hollow fibres using a mixture containing copper particles, polymer and solvent. This mixture was pressed through a spinneret into a coagulation bath. In this bath, non-solvent induced phase separation arrests the copper particles in the polymer matrix. Owing to the use of a bore liquid during spinning, hollow fibres were obtained. By thermal treatment, the polymer was decomposed and the copper particles sintered together, resulting in hollow, porous CuO fibres^30^. Hydrogenation of these CuO fibres at elevated temperatures was applied to convert CuO into metallic copper. The details of the physical characterization and X-ray diffraction patterns of the copper powder and the fibres can be found in Supplementary Fig. 1. A typical scanning electron microscope (SEM) image of the precursor Cu powder is shown in Supplementary Fig. 2 and of the hollow fibres in Fig. 1 (see Supplementary Fig. 3 for the locations at which these images were taken). The images of the external surface of the fibres show that the fibre is composed of aggregated copper particles forming an interconnected three-dimensional (3D) porous structure (Fig. 1a,b). The cross-sectional images of the deliberately broken fibres exhibit finger-like voids perpendicular to the surface that are terminated by a 10- to 15-μm-thick sponge-like porous outer layer (Fig. 1c,d). Cu hollow fibres have outer and inner diameters ranging from 1.55±0.1 to 1.3±0.05 mm, respectively (Fig. 1e). CO_2_ was purged from the inside out of the fibre, needing an overpressure of 1.70±0.1 bar due to the resistance of the porous structure. Gas bubbles emerging out of the fibre can be clearly seen in Fig. 1f (see also Supplementary Movie 1). The pressure is considered to drop evenly across the outer porous layer to 1.05 bar.

### Electrocatalytic performance of the copper hollow fibres

**Figure 2:**
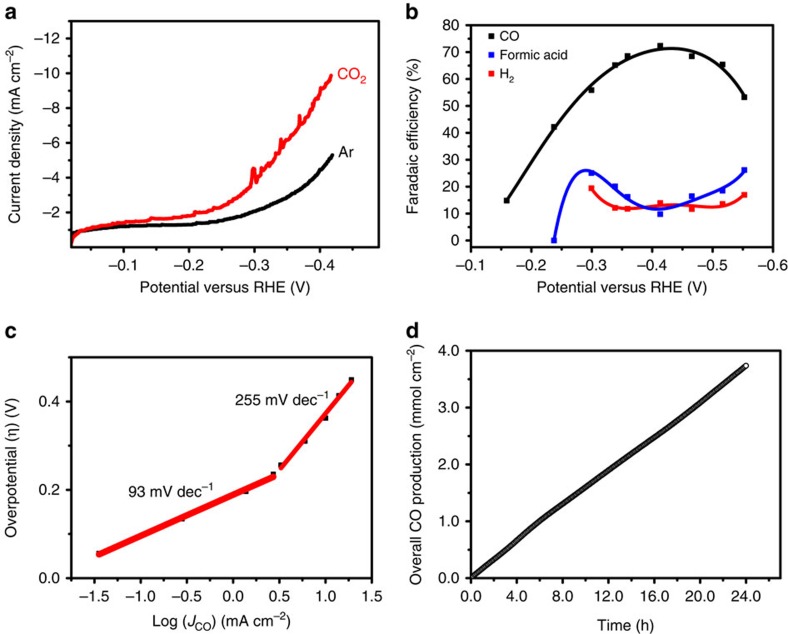
Electrocatalytic performance of the Cu hollow fibres. (**a**) Linear polarization curves obtained for Cu hollow fibres when CO_2_ or Ar was purged in 0.3 M KHCO_3_ electrolyte (scan rate: 50 mV s^−1^). (**b**) FE of CO, formic acid and H_2_ as a function of applied potential, using a CO_2_ purge of 20 ml min^−1^. (**c**) Overpotential versus partial current density of CO using Cu hollow fibres (flow rate of CO_2_: 20 ml min^−1^). (**d**) Total production of CO at an applied potential of −0.4 V for 24 h of continuous experiment (flow rate of CO_2_: 20 ml min^−1^).

Linear sweep voltammetry was performed in Ar or CO_2_ saturated electrolyte, while Ar or CO_2_ were purged through the fibres (Fig. 2a). The current densities recorded during Ar purge are due to evolution of hydrogen, which has an onset potential of around −0.25 V versus RHE. Purging of CO_2_ through the fibre wall leads to a *ca*. twofold increase in cathodic current density at potentials between −0.2 and −0.4 V versus RHE. This is contrary to the literature, in which for smooth or rough copper surfaces lower current densities were reported in CO_2_ atmosphere as compared with Ar atmosphere^31^,^32^. Extensive coverage of the Cu surface by CO, inhibiting the hydrogen evolution reaction, was proposed to explain the lower current density recorded during CO_2_ electroreduction on polycrystalline copper electrodes^33^. Thus, the high cathodic current achieved in the presence of CO_2_ in this study is already an indication of distinctive performance of Cu hollow fibres towards CO_2_ reduction.

The FE of the major products was measured by varying the applied potential between −0.15 and −0.55 V versus RHE (Fig. 2b). The onset of CO formation can be observed at −0.15 V versus RHE, implying an overpotential of just ≈40 mV above the equilibrium potential (−0.11 V versus RHE). The total FE efficiency of CO_2_ reduction products adds up to ≈85% at potentials between −0.3 and −0.5 V versus RHE. Specifically, a maximum FE of ≈72% was obtained towards CO at a potential of −0.4 V versus RHE, whereas in the literature a maximum FE for CO on polycrystalline copper and copper nanoparticles has been reported of only 20% (−0.8 V versus RHE) or 45% (*J*_CO_≈300 μA cm^−2^), respectively^19^,^20^. The decrease in FE of CO at more negative potentials (<−0.5 V; Fig. 2b) implies CO formation is most probably limited by desorption or consecutive surface reactions at these more negative potentials. Indeed, ethylene was detected (Supplementary Table 1) at these conditions, which is probably formed by coupling of two CO molecules^34^.

The electrokinetic data, represented by the Tafel plot, are shown in Fig. 2c. The first step in the process of CO_2_ activation involves electron transfer to adsorbed CO_2_, which is probably proton assisted^35^. Subsequently, the COOH intermediate reacts with a second electron and proton to form CO and water. A Tafel slope of around 116 mV dec^−1^ has been assigned to a mechanism in which formation of this COOH intermediate is rate determining in the formation of CO (refs 17, 20). The lower slope of 93 mV dec^−1^ observed in Fig. 2c is most probably due to a non-uniform potential or current distribution in the porous matrix of the hollow fibre. This might be caused by the extensive bubble formation and associated inhomogeneous distribution of reactants over the electrode surface. An apparent increase in Tafel slope can be observed in Fig. 2c at more negative potentials, which suggests a change in rate-determining step. At these potentials, hollow fibres show lower selectivity towards formic acid than typically reported in the literature for smooth or rough copper surfaces^19^,^20^, whereas similar to copper nanoparticles at high pressures^36^, a very high selectivity towards CO is still obtained. The increase in CO selectivity at higher potentials might be associated with the participation of another CO_2_ molecule in the rate-determining step of the mechanism^37^.

To test the stability of the Cu hollow fibres, 24 h of continuous electrolysis was performed at an applied potential of −0.4 V versus RHE (Fig. 2d and Supplementary Fig. 4). After a ≈10% drop in activity in the first 7 h, noticeable from the slight curvature of the plot in Fig. 2d, stable performance was achieved in the subsequent 17 h of experiment. SEM images of the morphology of the Cu hollow fibres before or after extensive electrolysis (Supplementary Fig. 5) did not show any differences. The activity of polycrystalline Cu typically diminishes very quickly (within an hour), unless very high purity electrolytes and electrodes (99.9999%) are employed^38^. It is important to note that studies using Cu plates usually discuss ultra-high purity copper, whereas the purity of the precursor copper powders used in this study is relatively low (99%), significantly reducing the price for commercial application. The observed stability of the hollow fibres is in agreement with that of Cu nanoparticles derived from Cu-oxide precursor films^20^.

**Figure 3:**
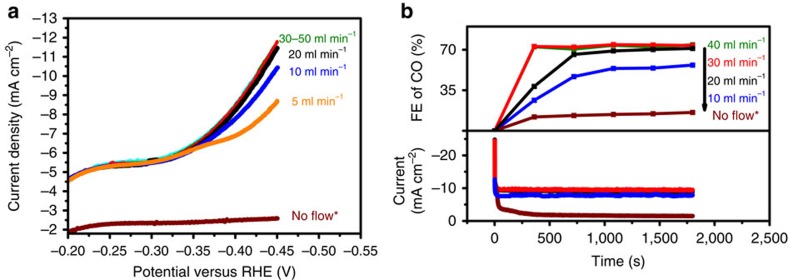
Electrocatalytic performance as a function of gas flow rate. (**a**) Linear polarization curves obtained at different gas flow rates of CO_2_ (scan rate 50 mV s^−1^). (**b**) FE towards CO for different flow rates of CO_2_ and the corresponding current densities (applied potential was −0.4 versus RHE and electrolyte concentration 0.3 M KHCO_3_). *The experiment was performed in CO_2_ saturated solutions.

Figure 3a,b show the effect of the CO_2_ flow rate on overall current density and FE of CO, respectively. The current density clearly depends on the CO_2_ flow rate up to 30 ml min^−1^, which yields a maximum FE of 75% at −0.4 V versus RHE for CO. This FE is almost twice of what has been recently reported for copper nanoparticles at the same potential^20^. The change in FE towards CO as a function of CO_2_ flow rate is consistent with a concurrent increase in current density. These experiments indicate that the FE of CO strongly depends on the efficiency of mass transfer of CO_2_ to the electrode surface. Similarly, reasonable activity and FE towards formic acid and CO (FE of 45% at −0.5 V versus RHE) were achieved using copper nanofoams prepared by electrodeposition^39^. The porous structure and thickness-dependent activity of nanofoams suggest that mass transfer phenomena inside the pores might play a role in improved selectivity towards CO_2_ electroreduction over hydrogen evolution. The steady behaviour above the flow rate of 30 ml min^−1^ implies most active sites are involved in converting CO_2_ to CO and the catalyst has reached its intrinsic limit. Previously, the CO_2_ reduction rate in aqueous conditions was shown to be proportional to the CO_2_ pressure applied, but optimized rates were not achieved even at pressures as high as 25 atm (ref. 40). As previously stated, this might be explained by a slow desorption rate of CO, a high CO surface coverage being supported by spectroscopic studies^17^. Therefore, the hollow fibre configuration might also be beneficial for (CO_2_ stimulated) removal of CO from the surface, induced by the very high local concentration of CO_2_ near the electrode surface.

**Figure 4:**
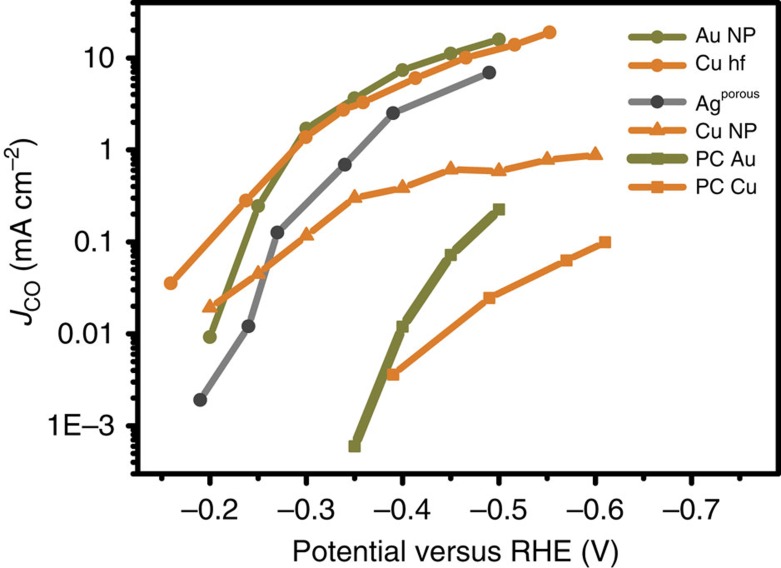
Activity of various electrodes in water. Comparison of the performance of different electrodes on the basis of the partial current density with CO at variable potentials. Data are derived from refs 9, 11, 20.

A comparison of the performance of our Cu hollow fibre to the performance of various electrodes, including those composed of other metals, is shown in Fig. 4. To this end, the partial current density of CO (*J*_CO_) is plotted against the applied potential. Figure 4 implies that Cu hollow fibres can reduce CO_2_ to CO electrochemically at a potential of −0.4 V versus RHE with over 15 to 400 times higher rate than polycrystalline Cu and Cu nanoparticles, respectively (see for reproducibility the Supplementary Fig. 6)^20^. Although outcompeting these currently best-performing copper-based electrodes, Cu hollow fibres also show comparable performance at low potentials (−0.2 to −0.6 V versus RHE) to that of noble metal catalysts evaluated in aqueous solutions (Au nanoparticles^11^ or nanoporous Ag^9^). Although noble metal electrodes benefit from a high overpotential for hydrogen evolution, Cu hollow fibres perform so well on the basis of the extraordinary efficient mass transfer of CO_2_.

## Discussion

Besides the aforementioned favourable mass transfer properties of the Cu hollow fibres, the remarkable formation of CO at very low overpotentials might also be associated with the nature of the copper sites obtained by hydrogen-induced reduction of CuO (refs 20, 41). The efficiency of rough and porous copper electrodes has been attributed to the oxide-derived formation of metastable copper sites existing in grain boundaries^42^. Furthermore, Reske *et al.*^43^ showed the reduction of CO_2_ to CO can be significantly enhanced by decreasing the size of the copper nanoparticles, which was correlated to the number of uncoordinated sites. The enhanced CO_2_ reduction observed at lower potentials on rough copper surfaces implies defect sites may be responsible for the increase in activity, which probably favour the formation of the COOH intermediate.

Analysis of the precursor copper powder and Cu hollow fibres, before and after electrolysis by X-ray photoelectron spectroscopy (XPS), indicate that major metal impurities at the surface are absent (Supplementary Fig. 7 and Supplementary Table 2). The major impurity is carbon, which is present in varying quantities in the copper powder used as precursor for synthesis, and hollow fibres before and after electrolysis. XPS spectra of the fibres further indicate that the surface, besides Cu^0^, contains some Cu_2_O, the latter likely to be associated with the exposure of the hollow fibre to air before introduction in the vacuum chamber of the XPS apparatus (Supplementary Figs 8 and 9). More importantly, the similarity in binding energy of the Cu 2*p* peaks before and after preparation or electrolysis suggests alloy or carbide formation on annealing or electrolysis is very unlikely, and that the activity is indeed associated with specific copper sites.

In addition to the activity comparison given in Fig. 4, comparison of the performance of hollow fibres and GDEs is useful, as the latter are typically used to induce electrochemical processes at gas–liquid–solid interfaces efficiently. Unfortunately, in most of the studies evaluating Cu-based GDE performance in CO_2_ reduction, high overpotentials (>1 V) have been applied, which are needed to stimulate hydrocarbon formation^44^,^45^. Although very high current densities (0.1 A cm^−2^ up to 1 A cm^−2^) have been reported at these potentials^8^,^46^, CO formation is usually not feasible, as evident from data reported for Ag-GDEs requiring an onset potential of around −0.6 V versus RHE, to initiate performance^47^. The thickness of the porous catalyst layer used in GDEs is typically in the range from 5 to 20 μm (ref. 48), similar to that of 15–20 μm determined for our Cu hollow fibres by electrodeposition of nickel and subsequent energy-dispersive X-ray analysis (see Supplementary Fig. 10 and Supplementary Methods for experimental details). This thickness is also comparable to the thickness of the oxide films used to prepare rough electrodes or electrodeposited 3D porous structures^49^. It should be noted that the geometrical current density of the fibres has been calculated by normalizing the current to the outer surface area of the cylindrical morphology, in agreement with the convention for reporting current densities of GDEs^8^,^50^,^51^.

Although GDEs play an important role in fundamental electrocatalysis, mass production is limited by economic and technical issues^48^. On the contrary, a mature dry–wet spinning process allows mass production of organic hollow fibres, which are already commercially available^52^. Furthermore, preparation of metal hollow fibres with diameters in the range of 100–500 μm was recently developed, implying great flexibility in the production of hollow fibres with variable diameters^30^,^53^. As a microtubular geometry has been applied in solid oxide fuel cells for some time, optimized stack design, sealing or current collection employed in this technology can be used to further optimize the configuration of metal hollow fibres when deployed for electrochemistry in liquid media^22^,^27^. Finally, we believe there is plenty of room to increase the electrochemical production rate by controlling the internal or external structure of the hollow fibres^24^,^54^,^55^. The thickness of the active catalyst layer can be tuned by changing the 3D geometry, the support material, the porosity and/or the precursor particle size.

In summary, the results reported in this article highlight a new electrode configuration to be explored for the development of robust electrolysis of CO_2_ at high rates in aqueous media. Employing a simple, compact Cu hollow fibre as both gas diffuser and cathode leads to very high CO production rates that are comparable to those achieved by use of noble metals. Selective formation of CO is observed with a maximum FE of 75% at a potential of 0.4 V versus RHE, when CO_2_ flow rates exceed 30 ml min^−1^. Partial current densities for CO_2_ reduction ranging from 2 to 17 mA cm^−2^ were obtained at moderate potentials (−0.3 to −0.5 V versus RHE). The remarkable electrocatalytic performance of the electrodes is attributed to a defect-rich porous structure in addition to extraordinary favourable mass transport conditions. Hollow fibre-based electrodes might generally be a promising solution to stimulate electrochemical reactions in which at least one gas-phase reactant with low solubility is involved.

## Methods

### Preparation of copper hollow fibres

Commercially available copper powder (Skyspring nanomaterials, 99%) with a particle size of 1–2 μm was used as catalyst precursor (see Supplementary Table 3 and Supplementary Fig. 9). N-methylpyrrolidone (NMP, 99.5 wt%, Sigma Aldrich) and Polyetherimide (PEI, Ultem 1,000, General Electric) were used as solvent and polymer, respectively. Copper powder (71.09 wt%) was added to NMP (22.14 wt%) followed by stirring and ultrasonic treatment for 30 min. After addition of PEI (6.76 wt%), this mixture was heated and kept at 50 °C and 60 °C for 30 min and 2 h, respectively. Next, the solution was allowed to cool down by stirring overnight, followed by degassing. Vacuum was applied for 90 min and the mixture was subsequently left overnight.

Spinning was carried out at room temperature (21±3 °C) using a stainless steel vessel, which was pressurized at 1 bar using nitrogen. The mixture was pressed through a spinneret (inner and outer diameters of 0.8 and 2.0 mm, respectively) into a coagulation bath containing tap water. Deionized water was pumped through the bore of the spinneret with a speed of 30 ml min^−1^ and the so-called air gap was set to 1 cm.

After spinning, the fibres were kept in a coagulation bath for 1 day to remove traces of NMP, followed by drying for 1 day. The green Cu hollow fibres were thermally treated at 600 °C for 3 h (heating rate and cooling rates: 60 °C h^−1^) in air to remove the PEI and induce sintering of the copper particles. The oxidized hollow fibres were reduced by hydrogenation at 280 °C for 1 h in gas flow of 4% H_2_ in balance gas Argon. Heating rates and cooling rates applied were 100 °C h^−1^. X-ray diffraction patterns were collected using a Bruker D2 Phaser X-ray diffractometer, equipped with a Cu-Kα radiation source and operated at 30 kV and 10 mA. SEM images were taken using a Phillips FEI XL30 FEG-ESEM or FEI Sirion HR-SEM. The locations of the taken images are shown in Supplementary Fig. 10. XPS were recorded using a Quantera SXM (Scanning XPS microprobe) spectrometer equipped with an Al Kα (1,486.6 eV) X-ray source. The source was operated at a 25-W emission power, beam size of 200 μm and pass energy of 224 eV. The resolution of the spectrometer was equivalent to 0.1 or 0.2 eV for high-resolution scans of elements or the overall survey spectra of the Cu fibres, respectively. Further details of the method used to quantify (relative) elemental composition can be found in the Supplementary Methods description.

### Electrochemical CO_2_ reduction

All solutions were prepared and all glasswares were cleaned by using deionized water (Millipore MilliQ, 18.2 MΩ). Electrochemical CO_2_ reduction activity of Cu hollow fibres was measured by using a three-electrode assembly in a glass cell at room temperature and pressure. A Princeton Applied Research VersaSTAT 3 potentiostat was used to control the potential. The cell compartment of the counter electrode, made of Pt mesh, was separated from the working electrode by using a Nafion 112 membrane (Sigma Aldrich). A Ag/AgCl (3 M NaCl, BASI) reference electrode was placed near the working electrode by using a Luggin capillary and all the potentials were converted to the RHE scale using the well-known Nernst relation. Voltage drop was measured before the electrolysis and after the experiments manually compensated for. Cu hollow fibres (4±0.5 cm long) were used as working electrode and gas diffuser. The fibres were sealed at the bottom by using epoxy glue and connected to the gas inlet of the cell. The cathodic compartment was filled with 100 ml, 0.3 M KHCO_3_ (99.95%, Sigma Aldrich) solution and purged with CO_2_ for at least 20 min before the start of the experiments. During the electrolysis, CO_2_ was purged continuously through the fibre at a rate of 20 ml min^−1^, unless otherwise indicated. The composition of the gas was sampled via gas chromatography every 6 min. CO, CO_2_, H_2_ and hydrocarbons were separated using two different columns (a ShinCarbon 2 m micropacked column and a Rtx-1 column). A thermal conductivity detector and flame ionization detector were used to perform the quantitative analysis of the gas-phase products. The time needed to reach steady-state concentration was ∼10 min; thus, the reaction was performed for at least 20 min at each experimental condition. A control experiment was conducted at −0.5 V versus RHE under argon atmosphere. CO was not detected in such experiment, showing that residues of the polymers used during preparation of the hollow fibres did not contribute to CO formation in the electrochemical CO_2_ reduction experiments. Liquid products formed during electrolysis were analysed by using HPLC. A Prominence HPLC set up of Shimadzu was used, equipped with an Aminex HPX 87-H column from Biorad^4^.

## Additional information

**How to cite this article:** Kas, R. *et al.* Three-dimensional porous hollow fibre copper electrodes for efficient and high-rate electrochemical carbon dioxide reduction. *Nat. Commun.* 7:10748 doi: 10.1038/ncomms10748 (2016).

## Supplementary Material

Supplementary Information Supplementary Figures 1-10, Supplementary Tables 1-3 and Supplementary Methods

Supplementary Movie 1 Copper hollow fiber employed as a working electrode. Applied potential is -0.4 V vs RHE.
