# Supplementary material for: Three-dimensional porous hollow fibre copper electrodes for efficient and high-rate electrochemical carbon dioxide reduction
Source: Nat Commun. 2016 Feb 18;7:10748. doi: 10.1038/ncomms10748 (PMC4759634; doi:10.1038/ncomms10748)
Supplement: Supplementary Information — Supplementary Figures 1-10, Supplementary Tables 1-3 and Supplementary Methods [file ncomms10748-s1.pdf]

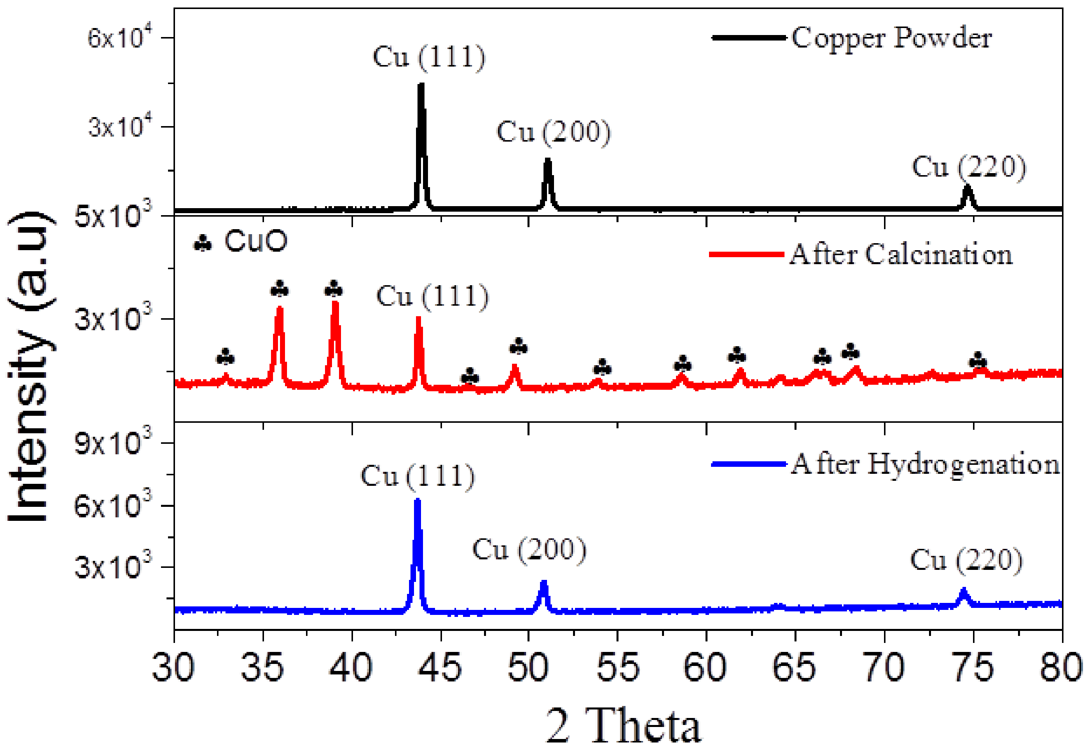

Supplementary Figure 1: XRD patterns of the parent copper powder, the Cu hollow fiber after calcination at 600 °C, and the copper hollow fiber after hydrogenation in H_2 (4% in balance gas Ar).

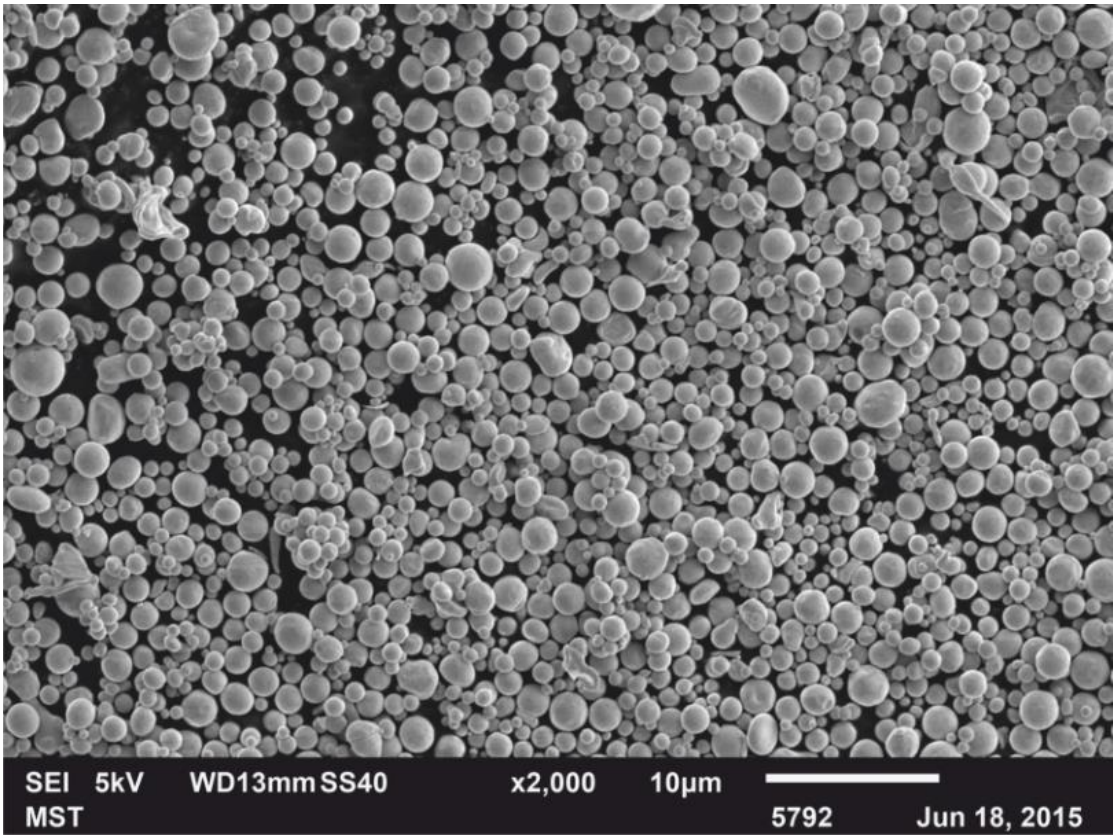

Supplementary Figure 2: SEM image of "as received" copper powder

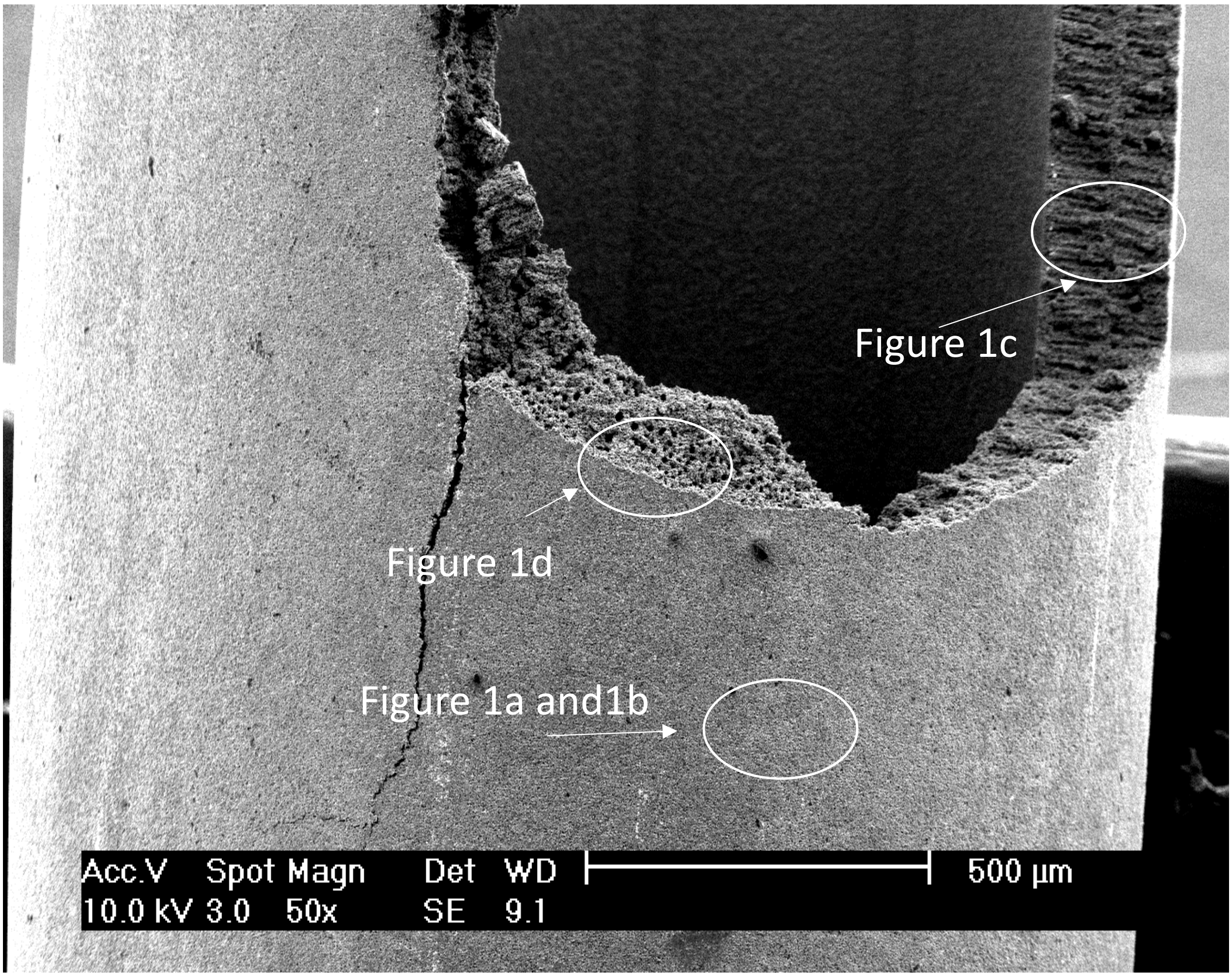

Supplementary Figure 3: SEM image of the Cu hollow fiber showing the locations at which the SEM images were taken to construct Figure 1 of the main article.

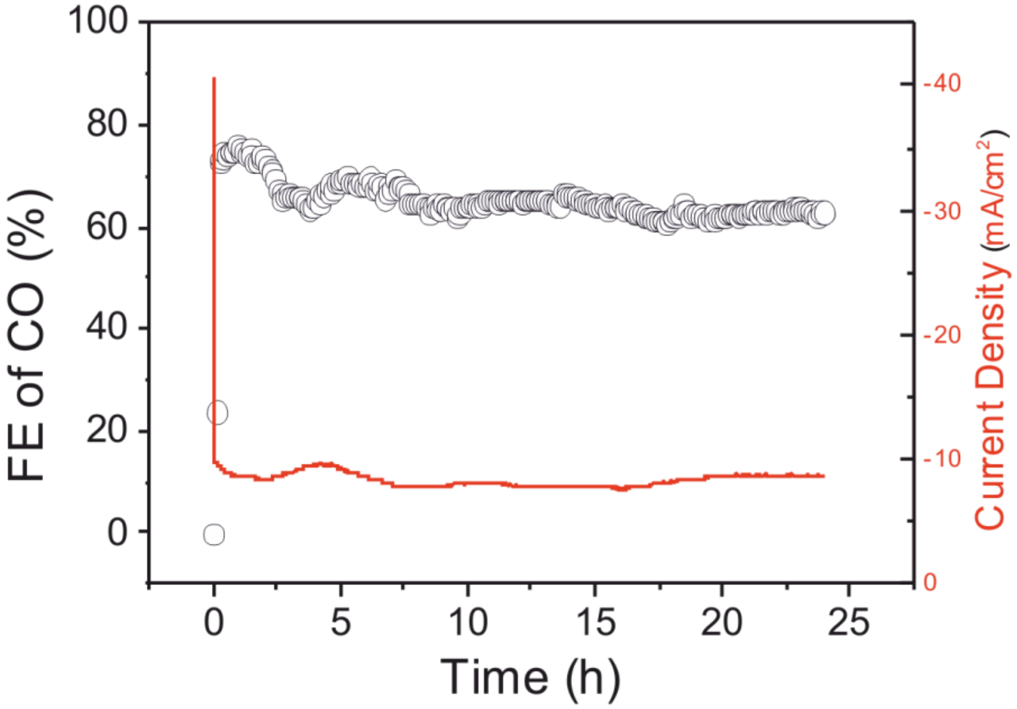

Supplementary Figure 4: The FE of CO and total current density at an applied potential of -0.4 V during 24 hours of continuous experiment (Flow rate of CO_2 : 20 ml min^{-1})

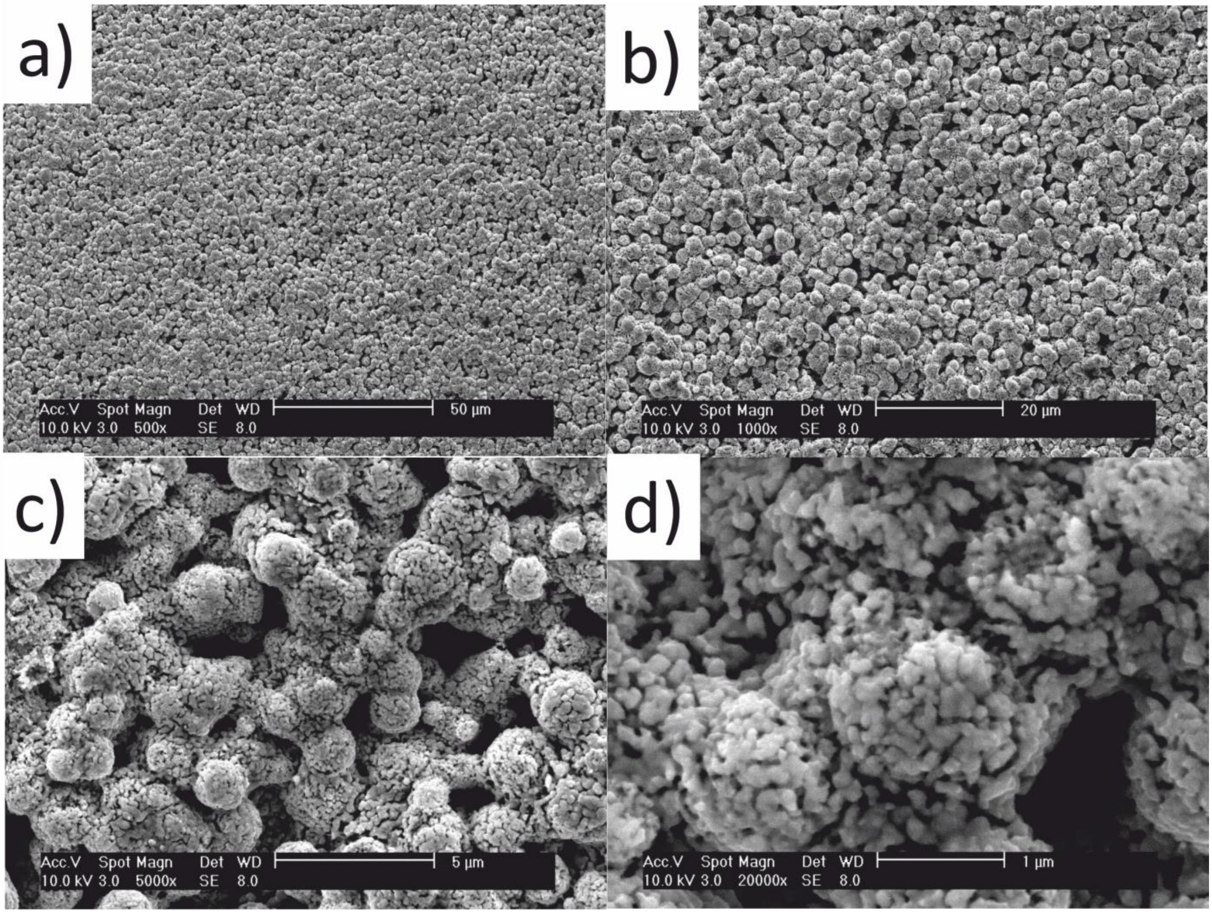

Supplementary Figure 5: SEM images of the Cu HF after 24 hours of electrolysis

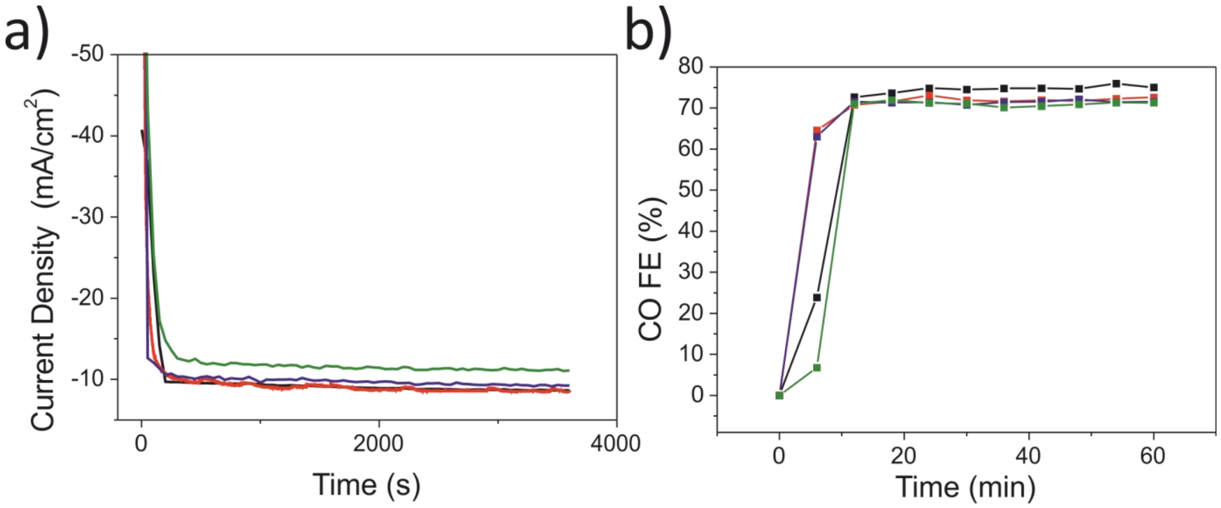

Supplementary Figure 6: Reproducibility tests with 4 different fibers at a potential of -0.4 V vs RHE. Flow rate of CO_2: 20 ml min^{-1}

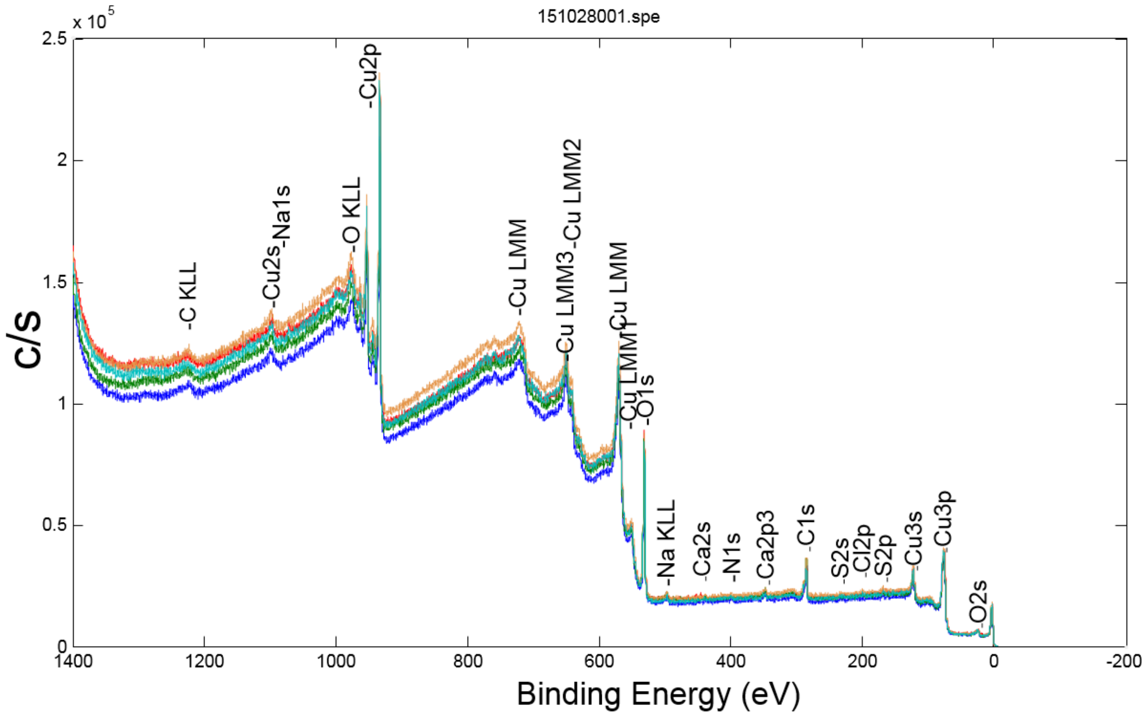

Supplementary Figure 7: X-ray photoelectron spectroscopy survey of copper hollow fibers before electrolysis (includes 5 repeated scans).

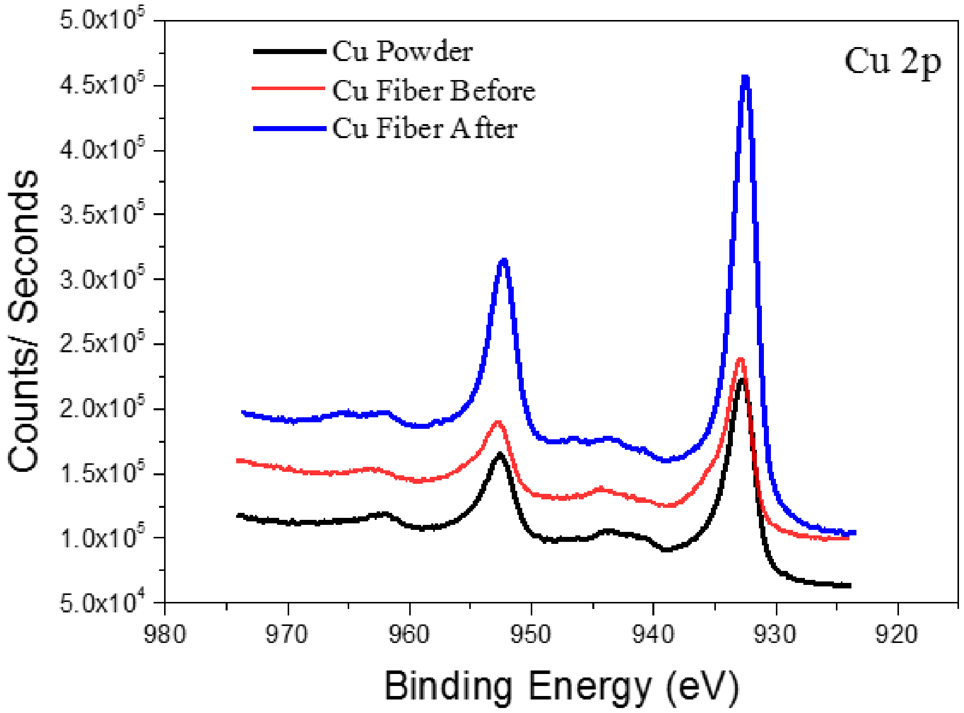

Supplementary Figure 8: High-resolution X-ray photoelectron spectra demonstrating the Cu 2*p* peaks indicative of predominantly Cu^0

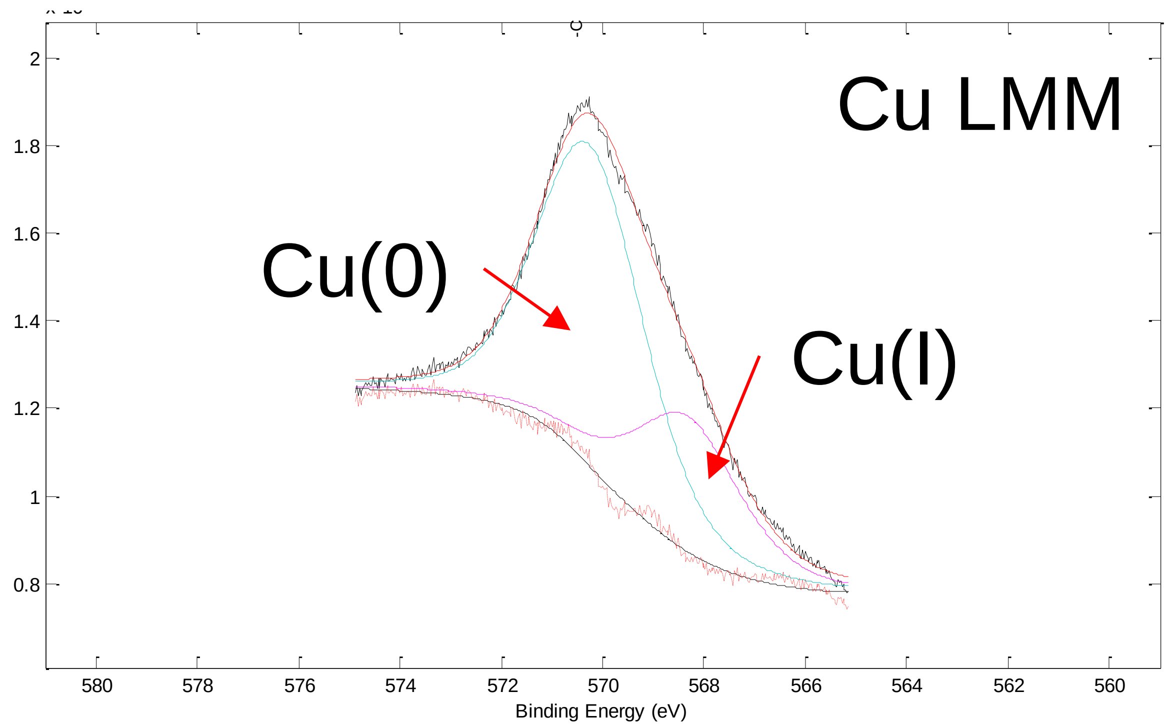

Supplementary Figure 9: High-resolution X-ray photoelectron spectrum of the Cu LMM region for Cu hollow fibers after electrolysis. Some Cu(I) might be present.

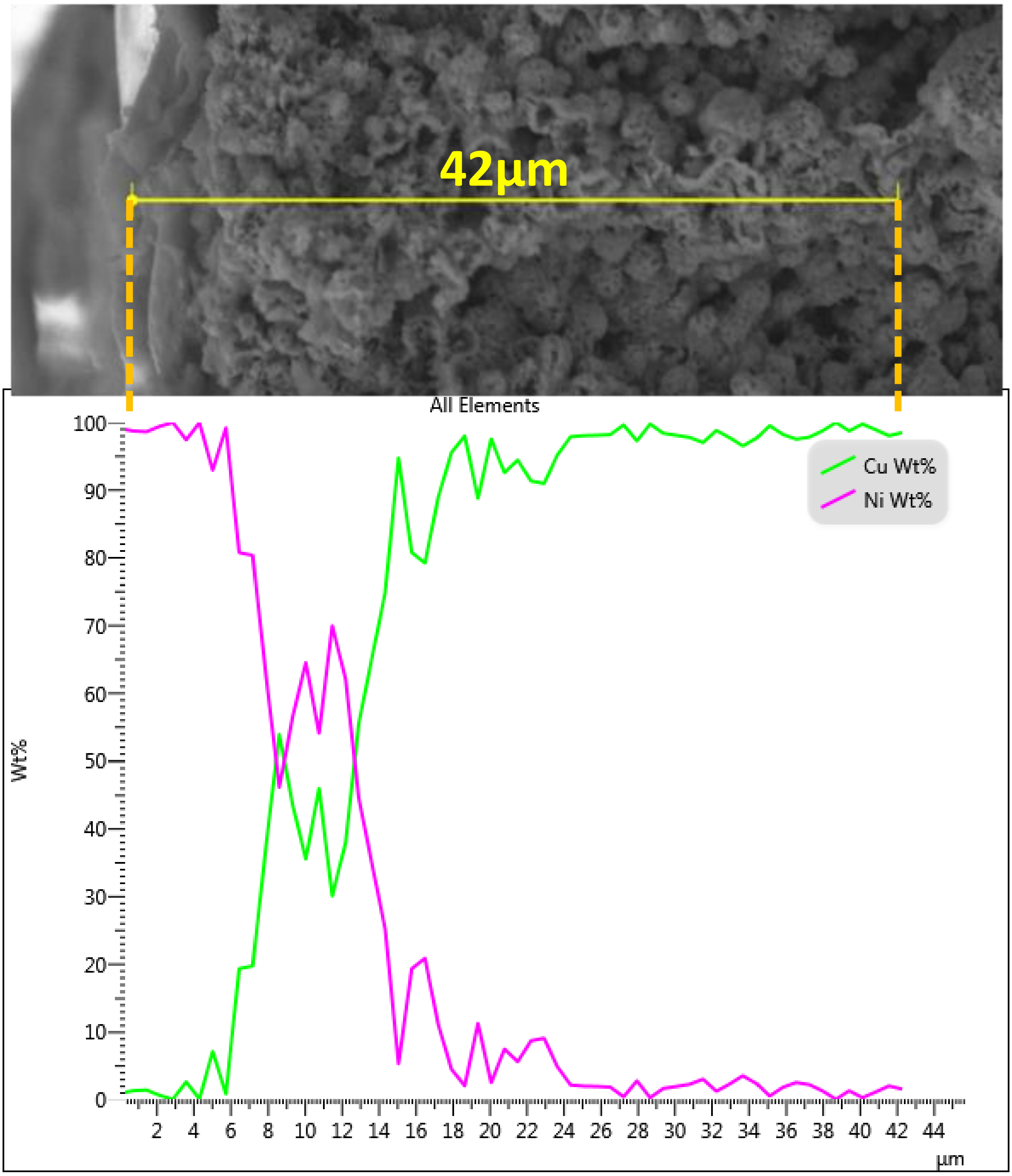

Supplementary Figure 10: EDX analysis demonstrating the wt% of electrodeposited nickel as a function of location, as measured from the outside inwards. Deposition of Ni was achieved on the copper hollow fibers feeding 20 ml min^{-1} of argon through the porous wall into a Ni^{2+} (nitrate) solution.

Supplementary Table 1: Summary of the faradaic efficiencies (FE) as a function of applied potential.

E vs RHE (V)	J_{total} (mA/cm^2)	Faradaic Efficiency (%)				
		CO	Formic Acid	Ethylene	H_2	Total
-0.16	0.24	14.8	0.0	0.0	-	14.8*
-0.24	0.66	42.2	0.0	0.0	-	42.2*
-0.30	2.5	55.9	25.1	0.0	19.4	100.4
-0.34	4.2	65.2	20.1	0.0	12.1	97.4
-0.36	4.8	68.6	16.2	0.0	11.8	96.6
-0.41	8.4	72.4	9.8	0.0	13.9	96.1
-0.47	14.7	68.5	16.5	0.1	11.7	96.8
-0.52	21.3	65.4	18.5	0.15	13.6	97.7
-0.55	35.7	53.3	26.2	0.2	16.9	96.6

* Hydrogen concentrations were not measurable at these low potentials due to the relatively low detection limit of the applied Thermal Conductivity Detector (TCD). The remaining current is considered to be due to formation of H_2, since formic acid was not detected at these potentials.

Supplementary Table 2: The atomic concentrations of the elements calculated from the intensities of the peaks present in XPS spectra.

Sample	C	N	O	Na	S	Cl	Ca	Cu	Cu^0/C^+ From Cu LMM fit
**Cu Powder**	9.39	-	43.87	-	-	-	-	46.74	21/79
**Cu Fiber (before)**	28.82	0.65	44.76	1.15	1.30	0.28	0.37	22.67	52/48
**Cu Fiber (after)**	7.53	-	42.40	-	-	-	-	50.06	73/27

Supplementary Table 3: Chemical analysis of the precursor copper powder as provided by Skyspring Nanomaterials.

Copper Powder		
**Purity**	99%	
**Average Particle Size**	1-2 µm	
**Contents**	C	5000
	O	10000
	Al	1000
	Ni	500
	Fe	500
	Pb	<100
	Cd	<100
	Hg	<10

Supplementary Methods

Nickel electrodeposition

Electrodeposition experiments were performed on Cu hollow fibers to determine the reactive zone. Nickel deposition was performed from solutions of nickel nitrate (50mM $Ni(NO_3)_2$, 5mA cm^{-2} for 900 s) while purging Ar at 20 ml min^{-1}. The SEM images in Supplementary Figure. 8 show that the Nickel deposition takes place mostly on the outer surface. Energy dispersive X-ray analysis (EDX) shows that the penetration depth of Nickel is around 15-20 µm which is indicative of the thickness of the boundary layer utilized during the reduction of CO_2. The noise in the line scan is due to the porous nature of the Cu hollow fiber. The current values obtained are normalized to the external (cylindrical) geometrical surface area of the Cu hollow fibers.

X-ray photoelectron spectroscopy measurements

X-ray photoelectron spectroscopy (XPS) was performed using a Quantera SXM (Scanning XPS microprobe) spectrometer equipped with an Al Kα (1486.6 eV) X-ray source. The source was operated with a 25 W emission power, beam size of 200 µm, and pass energy of 224 eV. For light elements, the detection limit was around 0.5 At-%, and for heavy elements around 0.1 At-%. The atomic concentrations of the elements measured were determined by the formula:

$$C_x = \frac{I_x/S_x}{\sum I_i/S_i}$$

in which I_x is the peak area of an XPS peak and S_x the relative sensitivity factor of the peak. The calculated amounts for various elements are presented in Supplementary Table 3. There is only a minor carbon impurity present at the surface of the Cu hollow fiber before electrolysis, which is most likely associated with the polymers used in the spinning process. This carbon impurity is no longer present after electrolysis. Furthermore, the Cu 2p XPS spectrum (Figure SI10) indicates the presence of Cu^0 and Cu^I. The latter is likely related to the formation of surface oxide by exposure of the sample to the laboratory atmosphere, before introduction in the vacuum chamber of the XPS apparatus.
